# Prevalence of overweight and metabolic syndrome, and associated sociodemographic factors among adult Ecuadorian populations: the ENSANUT-ECU study

**DOI:** 10.1007/s40618-020-01267-9

**Published:** 2020-05-19

**Authors:** J. Pérez-Galarza, L. Baldeón, O. H. Franco, T. Muka, H. A. Drexhage, T. Voortman, W. B. Freire

**Affiliations:** 1 Instituto de Investigación en Biomedicina (INBIOMED), Universidad Central del Ecuador, Quito, Ecuador; 2 Department of Epidemiology, Erasmus University Medical Center Rotterdam, Rotterdam, The Netherlands; 3 Department of Immunology, Erasmus University Medical Center Rotterdam, Rotterdam, The Netherlands; 4 Instituto de Investigación y Nutrición, Universidad San Francisco de Quito, Quito, Ecuador

**Keywords:** Overweight, Obesity, Metabolic syndrome, Demographics

## Abstract

**Background:**

Obesity and metabolic syndrome (MetS) are key risk factors for type 2 diabetes and cardiovascular disease. Little information exists on the prevalence of obesity and MetS in Latin America and specifically in Ecuador. We aimed to estimate the prevalence of overweight, obesity, and MetS among adults in Ecuador.

**Methods:**

We analyzed data from a nation-wide population-based survey in Ecuador (ENSANUT-ECU) among 10,318 participants (3684 men, 6634 women; age range: 18–59 years) conducted in 2012. Data related to residential location (urban versus rural), altitude (< 500, 500–1500 or > 1500 m above sea level (MASL)), region (highland, coast, amazon, or Galápagos), and socioeconomic status were collected. BMI, waist circumference, blood lipids, glucose, and blood pressure were measured by trained fieldworkers following standardized procedures.

**Results:**

The age-standardized prevalence of overweight was 39.5%; 22.3% was obese; and 31.2% had MetS. The prevalence of obesity, low HDL-cholesterol, and abdominal obesity were higher in women than in men, whereas men had a higher prevalence of hypertension (*p* < 0.05). Sex differences were not observed regarding the prevalence of combined MetS. Prevalence of both obesity and MetS was higher in urban areas, at low altitude regions (coast and Galapagos), and at high socioeconomic status (all *p* < 0.05).

**Conclusions:**

Prevalence of obesity and MetS in Ecuador are high. There are important demographic differences in the prevalence of MetS between Ecuadorian subpopulations that requires targeted research and prevention efforts, to hold and reduce the current public health problem of metabolic disorders.

**Electronic supplementary material:**

The online version of this article (10.1007/s40618-020-01267-9) contains supplementary material, which is available to authorized users.

## Introduction

There has been a dramatic epidemiologic transition from infectious diseases to non-communicable diseases in the past decades in developing countries [1]. This transition could be explained by the increased overweight prevalence [2, 3] which is associated with higher risk of developing metabolic syndrome (MetS), cardiovascular diseases (CVD), and diabetes mellitus type 2 (DM2), which are currently the leading causes of morbidity and death in Latin America and worldwide [4–6].

In Latin America, Mexico has the highest prevalence of cardiometabolic risk factors, with an estimated 67% of the population being overweight or obese [3], and 50% in the population older than 20 years having MetS [7]. Meanwhile, the prevalence of overweight and obesity is also high in other Latin-American countries, with estimates varying from 20 to 50% [3, 8], while the prevalence of MetS was recently estimated to be around 25% in the population between 18 and 65 years old, where low HDL-cholesterol and abdominal obesity were the most frequent components [7]. This high prevalence of MetS could be explained by the epidemiological transition, globalization, migration (from rural to urban areas), and corresponding changes in patterns of physical activity to a more sedentary life style, and shifts from diets based on natural or minimally processed foods and high in Andean grains and vegetables to a diet high in processed and ultra-processed foods and sugar-sweetened beverages [2, 9]. For Ecuador, there is not much information in a national context; data available so far are based only on the population of the capital city, Quito for which it was reported that 20% of women and 7.5% of men between 25 and 64 years of age have MetS [10]. In another study in a population older than 65 years in Quito, the prevalence of MetS was estimated to be 40% [11]. However, like other Andean nations in Latin America, Ecuador has a huge diversity with four different geographical regions (highlands, amazon, coast, and Galapagos Islands), each one with its own foods habits, altitude [from 0 to over 3000 m above sea level (MASL)], genetic background, economic status, and levels of urbanization [12]. Furthermore, there are several different ethnic groups in Ecuador with most being mestizos (Indian with European mix), 32 distinct Amerindian tribes, African descendants, Mulatos (African descendants with European mix), and Caucasian populations in low proportion [12]. A recent survey in China has shown a wide variability in MetS prevalence between rural and urban areas, and between regions [13], but whether this is also the case for Ecuador this is unknown. Consequently, it is important to establish the metabolic condition of Ecuadorian population taken in consideration socioeconomic status, geographical location, altitude, and ethnic origin that will allow a better estimation of the population burden of MetS and the implementation of new and more effective prevention strategies.

To achieve this goal, the Health Ministry of Ecuador conducted a large national health and nutrition survey (hereafter, ENSANUT-ECU), in which details on the socioeconomic status, nutrition, and health of the Ecuadorian population between the age of 0–59 years were evaluated.

Using data obtained in the ENSANUT-ECU survey, we aim to address the distribution and prevalence of overweight and MetS, and to study differences by gender, socioeconomic status (SES), ethnicity, and geographical location among the inhabitants of Ecuador.

## Methods

### Study population

The nation-wide cross-sectional population-based survey, ENSANUT-ECU, was based on a multi-stage, stratified sample design in nationally representative population aged 0–59 years in Ecuador [14, 15]. The population was stratified by rural and urban areas, regions, and provinces. For each province, 64 census tracts were initially preselected from which 12 occupied households were selected by simple random sampling. For 57,727 individuals from 19,803 households, information on sociodemographic characteristics and anthropometric measurements were collected. From a random subsample of 14,989 persons, a venous blood and urine specimens were collected.

The ENSANUT-ECU study was approved by the Institutional Review Board of the San Francisco de Quito University. All participants signed informed consent forms, and all data were pseudonymized during data entry and analysis [14].

For the current study, participants aged 18–59 years with complete information on sociodemographic and biochemistry data were selected. This resulted in a study population of 10,318 participants (3684 men and 6634 women). Data from 12 questionnaires were collected, from which for the current study, the following data were used: socioeconomic and demographic information; data related to location (urban or rural), and altitude [< 500, 500–1,500 or > 1,500 m above the see level (MASL)], region (highland, coast, amazon, or Galápagos). In addition, we used data that were collected from measurements: anthropometry, blood pressure; and the following biomarkers: total cholesterol, HDL-cholesterol, triglycerides, and fasting plasma glucose.

### Data collection

Applied structured questionnaires and measurements to participants in the selected households was performed by trained fieldworkers with the use of standardized procedures, protocols, and equipment [16]. Height was measured in subjects using portable stadiometers to the nearest 0.1 cm. Abdominal circumference was measured with standard tape measure to the nearest 0.1 cm. Portable electronic scales were used to measure weight in adults to the nearest 0.1 kg. Anthropometric data were collected twice for each variable with an interval of 5–10 min, to ensure reliability, and the mean of the two measurements was used. Blood pressure was measured twice using a digital sphygmomanometer. If there was a difference of ± 0.5 kg in weight, ± 0.5 cm in height or abdominal circumference, or ± 10 mmHg in blood pressure, a third measure was taken, and the two closest values were used to calculate the mean. To ensure the quality of the data, supervisors re-measured weight and height of participants in every tenth household, and interviewers were retrained every 11 days of fieldwork. National identity cards were used to verify participants’ age. Using the standardized procedures, venous blood and urine samples for biochemical determinations were collected [14]. Blood concentrations of total cholesterol, HDL-cholesterol, triglycerides, and glucose were measured using an automated enzymatic-colorimetric assay Modular Pre-Analytics Evo analyzer (Roche Diagnostics).

### Definitions of overweight, metabolic syndrome, and triads

BMI was calculated as weight in kilograms divided by the square of height in meters (kg/m^2^). Underweight was defined as a BMI < 18.5 kg/m^2^, overweight as BMI ≥ 25 and < 30 kg/m^2^, and obesity as BMI ≥ 30 kg/m^2^ [17]. MetS was defined according to the harmonized criteria of a joint interim statement of the International Diabetes Federation task force [18] as the presence of three or more of the following risk factors: elevated waist circumference (men ≥ 90, women ≥ 80 cm), elevated triglycerides (≥ 150 mg/dL), reduced HDL-C (≤ 40 mg/dL), elevated blood pressure (systolic ≥ 130 and/or diastolic ≥ 85 mm Hg), or elevated fasting glucose (≥ 100 mg/dL). In addition to MetS, we defined combinatory dangerous triads, which have been shown to be associated with higher CVD and overall mortality, as the combination of abdominal obesity, high blood pressure, and hyperglycemia and the combination of low HDL, high blood pressure, and hypertriglyceridemia [6].

### Sociodemographic factors

For obtaining an approximate index of economic welfare, a factor analysis was used based on 42 variables. The variables used in this procedure were those related to home ownership, household equipment, and characteristics of housing variables, such as ownership of kitchen equipment, laptop, desktop, conventional television, led television, or a DVD player, on which information was obtained from the questionnaires. Once estimated, the model builds a score by means of a regression. With this technique, an index of economic welfare was obtained, which summarizes the structure of variance and covariance of all the 42 variables included. The score was estimated at the level of the household. A higher score represents a better economical position for that household. We categorized the score into quintiles that were then used as a categorical variable. Those households in the lowest quintile (Q1) are those households with the poorest economic conditions in terms of characteristics of housing and holding assets, while those in the highest quintile (Q5) represent households with better economic conditions [14].

**Fig. 1:**
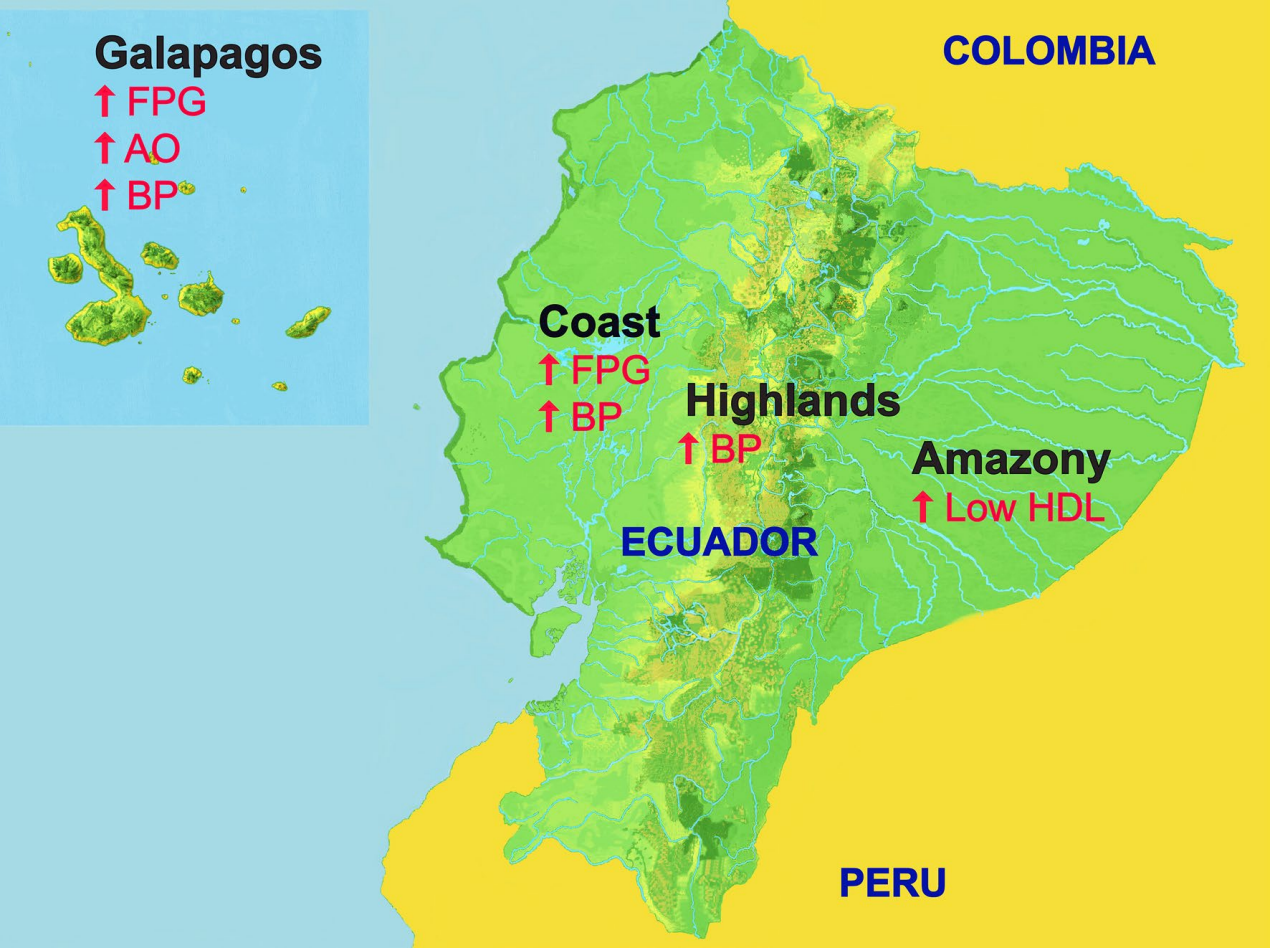
Figure of the map of Ecuador that summarizes the significant metabolic abnormalities on each region. All the metabolic abnormalities highlighted on each region are significant against at least another region (*p* < 0.05)

The altitude of the households was determined by GPS devices and was categorized into meters above sea level (MASL). Ecuador has four regions: the coast region is located between the Pacific Ocean by the east and the Andes mountains by the west; the highlands correspond to the Andean mountains that cross Ecuador from South to North and divided the territorial land in three regions; the Amazon region is founded between the east border of the Andes mountains and the borders of Colombia and Peru in the west; and the Galapagos Islands that is the archipelago de Galapagos (see Fig. 1, which summarizes the metabolic abnormalities of each region). The coast and the Galapagos represent 3222 of 3279 inhabitants at 0–500 MASL region, 1333 individuals of the Amazon also belong to the 2114 subjects from 501–1500 MASL, and the 4037 participants from the highlands are also part of the group of 4925 living > 1501 MASL. The capitals of the provinces and the head of the cantons (Provinces are divided in cantons) are considered urban areas.

### Statistical analysis

Both crude and age-standardized prevalence of overweight, obesity, and Mets were calculated using the overall 2010 population census distribution for Ecuador [19]. We stratified age standardization by gender to be able to compare men and women. Data are presented as percentages, means with SDs, or medians with range, depending the type of analysis. We provide the estimates of the prevalence of overweight, obesity, and Mets in our whole-study population and stratified by gender, age, area of residence, region, altitude, ethnicity, and socioeconomic status. For the age-stratified analysis, we split the population into four age groups: 18–29, 30–39, 40–49, and 50–59 years old. We compared differences between groups using Chi-square tests, *t* tests, and ANOVA depending the nature of the variable analyzed, and we examined correlations with Spearman’s test. Figures were performed using GraphPad Prism version 5.00 for Windows, GraphPad Software, San Diego, California, USA.

## Results

### General description of the study population

Table 1 describes the general characteristics of the study population stratified by the presence or absence of MetS and by sex. As expected, age, weight, waist circumference, BMI, lipids, and fasting plasma glucose were on average higher in the population with MetS.

**Table 1:** Characteristics of participants by gender and by metabolic syndrome status

	Metabolic syndrome			
	Men without MetS 68.5% (2523)	Men with MetS 31.5% (1161)	Women without MetS 69.2% (4588)	Women with MetS 30.8% (2046)
Age (years)^c^ (10,318)	30 (41)	38 (41)	32 (41)	39 (41)
**Age groups**^**b**^ **(10,318)**				
18–29 (3711)	82.6% (1217)	17.4% (257)	84.8% (1897)	15.2% (340)
30–39 (3334)	64.4% (709)	35.6% (392)	68.4% (1527)	31.6% (706)
40–49 (2540)	53.9% (435)	46.1% (372)	55.0% (953)	45.0% (780)
50–59 (733)	53.6% (162)	46.4% (140)	49.0% (211)	51.0% (220)
Weight (kg)^a^ (10,318)	66.7 (10.9)	79.9 (11.8)	60.3 (11.3)	69.7 (12.1)
Height (m)^a^ (10,318)	1.7 (0.1)	1.7 (0.1)	1.5 (0.1)	1.5 (0.1)
Waist circumference (cm)^a^ (10,318)	84.7 (9.5)	98.5 (9.1)	84.6 (10.4)	94.9 (9.9)
SBP (mmHg)^a^ (10,318)	118.5 (10.7)	128.1 (14.0)	111.4 (11.2)	122.3 (15.8)
DBP (mmHg)^a^ (10,318)	73.5 (8.5)	80.7 (9.9)	69.2 (8.1)	76.0 (10.3)
Total cholesterol (mg/dL)^a^ (10,318)	174.0 (36.5)	192.0 (38.0)	171.3 (35.9)	190.0 (36.6)
LDL-cholesterol (mg-dL)^a^ (10,085)	105.5 (31.6)	115.4 (33.8)	104.2 (29.8)	115.1 (32.5)
HDL-cholesterol (mg/dL)^a^ (10,318)	45.8 (11.0)	34.4 (7.1)	48.4 (12.1)	38.3 (7.9)
Triglycerides(mg/dL)^c^ (10,318)	101 (374)	199 (351)	88 (349)	173 (365)
Glucose (mg/dL)^a^ (10,318)	86.9 (11.5)	100.7 (37.1)	86.2 (11.0)	102.0 (38.8)
BMI (kg/m^2^)^a^ (10,318)	24.5 (3.5)	29.2 (3.6)	26.0 (4.4)	30.2 (4.8)
**BMI groups (Kg/m**^**2**^**)**^**b**^ **(10,318)**				
Underweight (< 18.5)	96.5% (55)	3.5% (2)	97.8% (90)	2.2% (2)
Normal (18.5–25)	93.5% (1427)	6.5% (100)	90.6% (1983)	9.4% (206)
Overweight (25–30)	58.2% (891)	41.8% (640)	65.1% (1712)	34.9% (916)
Class I obesity (30–35)	26.5% (126)	73.5% (350)	50.3% (633)	49.7% (626)
Class II/III obesity (35–40)	26.1% (24)	73.9% (68)	36.6% (169)	63.4% (293)
**Ethnic origin**^**b**^** (10,318)**				
Indigenous (952)	84.5% (277)	15.5% (51)	76.6% (478)	23.4% (146)
Mestizo (8377)	66.5% (1971)	33.5% (992)	68.1% (3685)	31.9% (1729)
Others (989)	70.0% (275)	30.0% (118)	71.3% (425)	28.7% (171)
**Altitude**^**b**^ **(10,318)**				
0–500 m (4372)	64.2% (1043)	35.8% (581)	67.0% (1840)	33.0% (908)
501–1500 m (1819)	71.3% (427)	28.7% (172)	73.2% (893)	26.8% (327)
> 1500 m (4127)	72.1% (1053)	27.9% (408)	69.6% (1855)	30.4% (811)
**Sub regions**^**b**^** (10,318)**				
Highlands (4925)	71.1% (1240)	28.9% (504)	69.5% (2210)	30.5% (971)
Coast (2957)	63.7% (722)	36.3% (412)	65.9% (1201)	34.1% (622)
Amazon (2114)	72.0% (502)	28.0% (195)	74.0% (1049)	26.0% (368)
Galapagos (322)	54.1% (59)	45.9% (50)	60.1% (128)	39.9% (85)
**Area**^**b**^** (10,318)**				
Urban (6565)	65.1% (1559)	34.9% (837)	67.6% (2818)	32.4% (1351)
Rural (3753)	74.8% (964)	25.2% (324)	71.8% (1770)	28.0% (695)

*SBP* systolic blood pressure, *DBP* diastolic blood pressure, *FPG* fasting plasma glucose

^a^Data are mean (standard deviation), ^b^Data are in percentage (number), ^c^Data are in median (range)

### Overweight, obesity and MetS by age, sex, and ethnic origin.

**Fig. 2:**
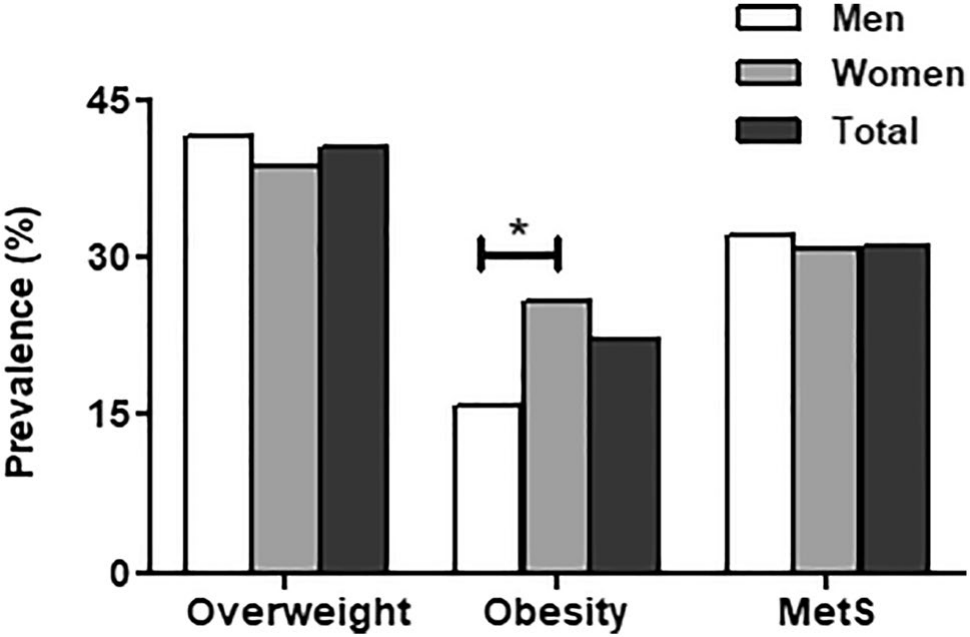
Age-standardized prevalence of overweight, obesity, and metabolic syndrome (MetS) in adults aged 18–59 in Ecuador. *p < 0.001

Overweight or obesity was present in 57% of men and in 65.7% of women (Fig. 2). This higher prevalence among women as compared to men was mainly driven by a higher prevalence of obesity (BMI > 30) (25.9% versus 15.4% in men, *p* < 0.001) (Fig. 2). Despite the higher prevalence of overweight and obesity in women, there was no significant difference in MetS between genders, with 31.5% of men and 30.8% of women having MetS (Fig. 2). MetS prevalence was higher in older adults and was three times higher in the age groups 40–49 and 50–59 (45.4% and 49.1%, respectively), in comparison with the age group of 18–29 years (16.1%) (Table 1). The prevalence of MetS was twice higher in mestizos and other ethnic groups than the indigenous population (Table 1).

### MetS by sex

Table 2 shows that men had a lower prevalence of abdominal obesity, low HDL-cholesterol, and overweight and obesity, whereas women had lower prevalence of hypertriglyceridemia and hypertension. The age-standardized prevalence of the cumulative numbers of the metabolic abnormalities of the MetS shows that only one-third of the female population have no abnormalities compared to one quarter of the male population. In line with this, men more often had more abnormalities than women (Table 3) (see Table, Online Resource 1, which illustrates the age-standardized prevalence of the accumulative risk factors of the metabolic syndrome by subregion, area, altitude, region, and socioeconomic quintiles and by gender).

**Table 2:** Crude and age-standardized prevalence of the metabolic syndrome

MetS	
Crude	35.9 (34.6–37.1)
**Gender**	
Men	32.1 (30.3–33.9)
Women	30.8 (29.4–32.1)
**Area**	
Urban	33.7 (32.2–35.1)*
Rural	27.0 (25.3–28.6)
**Altitude**	
0–500	34.1 (32.3–35.8)*
501–1500	27.4 (25.0–29.8)
> 1500	29.5 (27.9–31.2)
**Subregion**	
Highland	29.9 (28.4–31.5)
Coast	35.0 (32.8–37.1)*
Amazon	26.6 (24.4–28.8)
Galapagos	41.9 (34.9–49.0)*
**Economic quintile**	
Q1	23.6 (21.6–25.6)
Q2	30.4 (28.1–32.7)*
Q3	34.3 (31.8–36.8)*
Q4	35.9 (33.2–38.6)*^a^
Q5	32.4 (29.7–35.0)*

Results are age-standardized rate (95% CI)

*Significant differences (*p* value < 0.05) compared to rural, 501–1500 and > 1500, Highland and Amazon, and to Q1. ^a^Significant difference of Q4 compared to Q2

**Table 3:** Crude and age-standardized prevalence of the individual components of the metabolic syndrome and overweight and obesity

Individual components of the metabolic syndrome						
	Abdominal obesity	Hypertriglyceridemia	Low HDL cholesterol	High blood pressure	Hyperglycemia	BMI > 25 kg/m^2^
Crude	69.2 (67.5–70.8)	34.3 (33.1–35.5)	59.8 (58.4–61.3)	22.8 (21.8–23.8)	18.9 (18.0–19.9)	66.5 (64.9–68.1)
**Gender**						
Men	46.6 (44.4–48.9)	39.5 (37.5–41.5)^¶^	45.9 (43.7–48.1)	27.1 (25.4–28.8)^¶^	16.4 (15.0–17.7)	57.4 (54.9–59.8)
Women	74.0 (71.9–76.0)^β^	26.2 (24.9–27.4)	67.9 (65.9–69.9)^β^	14.6 (13.6–15.6)	15.2 (14.2–16.1)	64.6 (62.6–66.5)^β^
**Area**						
Urban	66.0 (64.0–68.0)^¶^	33.0 (31.6–34.4)^¶^	60.7 (58.9–62.6)	20.5 (19.3–21.6)^¶^	17.2 (16.2–18.2)^¶^	65.0 (63.0–66.9)^¶^
Rural	60.2 (57.8–62.7)	27.1 (25.4–28.7)	58.1 (55.7–60.5)	16.9 (15.6–18.3)	13.0 (11.8–14.2)	56.6 (54.2–59.0)
**Altitude**						
0–500	65.6 (63.2–68.0)	30.6 (29.0–32.2)	60.2 (57.9–62.5)	21.2 (19.8–22.6)^‡^	20.4 (19.0–21.7)^‡^	63.3 (60.9–65.6)
501–1500	64.0 (60.4–67.7)	29.6 (27.1–32.1)	65.4 (61.7–69.1)^‼^	11.3 (9.8–12.9)	11.0 (9.5–12.5)	62.6 (59.0–66.2)
> 1500	63.2 (60.8–65.7)	31.6 (29.9–33.3)	58.5 (56.2–60.8)	17.7 (16.4–19.0)^α^	10.6 (9.7–11.6)	61.9 (59.5–64.3)
**Subregion**						
Highland	63.8 (61.5–66.0)	31.9 (30.4–33.5)	58.8 (56.6–60.9)	17.7 (16.5–18.8)^+^	11.3 (10.3–12.2)	62.2 (60.0–64.4)
Coast	65.2 (62.2–68.1)	30.6 (28.6–32.6)	58.3 (55.5–61.0)	23.3 (21.5–25.0)*	23.1 (21.4–24.8)*	62.8 (60.0–65.7)
Amazon	62.9 (59.5–66.3)	28.7 (26.4–31.0)	66.6 (63.1–70.1)^π^	10.9 (9.5–12.3)	9.1 (7.8–10.4)	61.5 (58.2–64.9)
Galapagos	76.4 (66.9–85.9)*	29.8 (23.9–35.8)	65.2 (56.4–74.0)	22.4 (17.2–27.5)^+^	30.8 (24.7–36.8)*	74.8 (65.4–84.3)*
**Economic status quintile**						
Q1	55.6 (52.5–58.6)	23.9 (21.8–25.9)	55.9 (52.8–59.0)	16.2 (14.5–17.8)	13.1 (11.6–14.6)	53.3 (50.2–56.3)
Q2	65.7 (62.4–69.0)^‡^	29.5 (27.3–31.7)^‡^	61.1 (57.9–64.3)	17.5 (15.8–19.2)	14.3 (12.7–15.8)	62.8 (59.5–66.0)^‡^
Q3	66.5 (63.0–70.0)^‡^	33.0 (30.5–35.5)^‡^	63.3 (59.9–66.7)^‡^	18.7 (16.9–20.6)	15.7 (14.0–17.4)	64.1 (60.7–67.5)^‡^
Q4	68.5 (64.8–72.1)^‡^	35.1 (32.5–37.8)^‡^	62.7 (59.1–66.2)^‡^	19.5 (17.5–21.4)	16.9 (15.1–18.7)^‡^	68.4 (64.7–72.0)^‡^
Q5	67.1 (63.2–70.9)^‡^	34.1 (31.3–36.8)^‡^	59.6 (56.0–63.2)	18.8 (16.7–20.8)	14.5 (12.7–16.3)	66.4 (62.6–70.2)^‡^

Results are age-standardized rate (95% CI). Significant differences (*p* value < 0.05) compared to men^β^, women^¶^, rural^¶^, 501–1500 and > 1500^‡^, > 1500^‼,^ 501–1500^α^, Amazon^+^, Highland and Amazon*, Highland and Coast^π^, and to Q1^‡^

### Overweight, obesity and MetS in urban versus rural areas

The population living in urban areas had higher MetS (33.7%) than those in rural areas (27.0%) (*p* < 0.001). This difference between urban and rural areas was especially pronounced for men (34.9% of men in urban versus 25.2% of men in rural areas) (Table 1). The prevalence of overweight and obesity and of all MetS components, except for low HDL, was higher in urban populations. These differences were again mainly based on differences in men living in urban versus rural areas, while urban versus rural women differed to a smaller extent (see Table, Online Resource 2, which describes the age-standardized prevalence of the individual components of the metabolic syndrome and overweight and obesity by area, altitude, region, and socioeconomic quintiles and by gender). In line with this, urban populations had higher prevalence of 3, 4, and 5 abnormalities, again mainly explained by differences in men (Table 3) (see Table, Online Resource 2, which describes the age-standardized prevalence of the individual components of the metabolic syndrome and overweight and obesity by area, altitude, region, and socioeconomic quintiles and by gender).

### Overweight and MetS by altitude

Population that lived 0–500 MASL, had the highest prevalence of MetS (*p* < 0.001), hypertension (*p* < 0.001) and hyperglycemia (*p* < 0.001) of all altitude regions (Tables 2 and 3), whereas those living 501–1500 MASL had the highest prevalence of low HDL (*p* < 0.001) and the lowest prevalence of hypertension, especially in women (see Table, Online Resource 2, which describes the age-standardized prevalence of the individual components of the metabolic syndrome and overweight and obesity by area, altitude, region, and socioeconomic quintiles and by gender). The population living 501–1500 MASL had a significant higher prevalence of two metabolic abnormalities, while the population living 0–500 MASL had significant higher prevalence of four abnormalities both in men and women (Table 4).

**Table 4:** Crude and age-standardized prevalence of one or more components of the metabolic syndrome

Accumulative risk factors of the metabolic syndrome						
	0	1	2	3	4	5
Crude	13.2 (12.6–13.9)	22.3 (21.5–23.2)	28.6 (27.5–29.6)	21.1 (20.2–22.0)	11.7 (10.9–12.4)	3.1 (2.7–3.5)
**Gender**						
Men	25.2 (23.6–26.8)^¶^	22.8 (21.3–24.3)	19.9 (18.5–21.4)	18.2 (16.8–19.6)	11.2 (10.1–12.3)^¶^	2.7 (2.1–3.2)
Women	10.2 (9.4–11.0)	25.5 (24.2–26.7)	33.6 (32.2–35.0)^π^	20.0 (18.9–21.0)	8.6 (7.9–9.3)	2.2 (1.8–2.6)
**Area and altitude**						
Urban	14.7 (13.8–15.6)	23.1 (21.9–24.3)	28.5 (27.3–29.8)	20.3 (19.2–21.3)^ỻ^	10.5 (9.7–11.3)^ỻ^	2.9 (2.4–3.3)^ỻ^
Rural	17.8 (16.4–19.1)^β^	26.7 (25.1–28.4)^β^	28.5 (26.8–30.2)	17.8 (16.5–19.1)	7.7 (6.8–8.6)	1.5 (1.1–1.8)
**Altitude**						
0–500	14.5 (13.4–15.7)	23.5 (22.0–24.9)	27.9 (26.3–29.5)	20.3 (19.0–21.6)	11.1 (10.1–12.0)^‡^	2.7 (2.2–3.2)^α^
501–1500	16.2 (14.3–18.0)	23.6 (21.3–25.8)	32.9 (30.2–35.5)^‼^	18.8 (16.8–20.8)	7.1 (5.9–8.3)	1.4 (0.9–2.0)
> 1500	16.8 (15.5–18.1)	25.8 (24.3–27.4)	27.9 (26.3–29.5)	19.8 (18.4–21.1)	7.9 (7.1–8.8)	1.8 (1.4–2.2)
**Subregion**						
Highland	16.8 (15.7–18.0)^δ^	25.1 (23.7–26.5)^δ^	28.2 (26.7–29.7)	19.7 (18.4–20.9)	8.4 (7.6–9.2)	1.9 (1.5–2.3)
Coast	14.4 (13.0–15.7)	23.6 (21.9–25.4)	27.1 (25.2–28.9)	20.3 (18.6–21.9)	11.6 (10.3–12.8)^Δ^	3.1 (2.5–3.8)^Δ^
Amazon	15.9 (14.2–17.5)	25.1 (23.0–27.3)^δ^	32.4 (30.0–34.8)^±^	19.2 (17.4–21.1)	6.3 (5.2–7.4)	1.1 (0.6–1.5)
Galapagos	10.9 (7.3–14.5)	17.7 (13.1–22.3)	29.5 (23.6–35.4)	22.7 (17.5–27.9)	16.5 (12.0–20.9)^Δ^	2.8 (1.0–4.6)
**Economic status**						
Q1	19.7 (17.9–21.6)*	28.3 (26.1–30.5)^ϕ^	28.3 (26.1–30.5)	16.1 (14.4–17.7)	6.3 (5.2–7.3)	1.3 (0.8–1.7)
Q2	15.2 (13.6–16.8)	24.6 (22.6–26.7)	29.8 (27.6–32.1)	19.5 (17.7–21.3)	9.1 (7.9–10.4)^‡^	1.8 (1.2–2.3)
Q3	14.3 (12.6–15.9)	22.6 (20.6–24.7)	28.8 (26.5–31.1)	22.3 (20.3–24.3)^‡^	9.9 (8.6–11.3)^‡^	2.1 (1.4–2.7)
Q4	14.2 (12.5–15.8)	22.1 (20.0–24.2)	27.8 (25.4–30.1)	22.0 (19.9–24.1)^‡^	10.6 (9.2–12.1)^‡^	3.3 (2.5–4.1)^κ^
Q5	14.8 (13.0–16.6)	23.8 (21.5–26.1)	29.0 (26.5–31.6)	19.8 (17.8–21.9)^‡^	10.1 (8.6–11.5)^‡^	2.5 (1.8–3.2)^‡^

Results are age-standardized rate (95% CI). Significant differences (*p* value < 0.05) compared to women^¶^, men^π^, urban^β^, rural^¶^, 501–1500 and > 1500^‡^, 0–500 and > 1500^‼^, 501–1500^α^, Galapagos^δ^, Highland and Coast^±^, Highland and Amazon^Δ^, Q2–Q5*, Q3 and Q4^ϕ^, Q1^‡^ and to Q1 and Q2^κ^

### Overweight and MetS by region

**Fig. 3:**
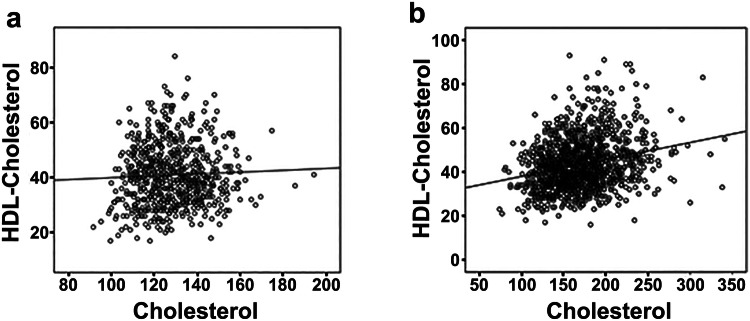
Correlation of total cholesterol and HDL. **a** In men, Spearman’s correlation coefficient 0.04, *p* 0.24, and **b** in women, Spearman’s correlation coefficient 0.26, *p* < 0.001

In line with the results for the low altitude, a higher prevalence, both in men and women, of MetS, hypertension, and hyperglycemia were found in the coast and in the Galapagos regions, as compared to the other regions (Tables 2 and 3). Also, prevalence of overweight or obesity was higher in the Galapagos region (74.8%) than in the other regions (61.5–62.8%) (Fig. 3, Table 3) (see Table, Online Resource 2, which describes the age-standardized prevalence of the individual components of the metabolic syndrome and overweight and obesity by area, altitude, region, and socioeconomic quintiles and by gender). The coast and the Galapagos also had the highest prevalence of four and five cumulative abnormalities (Table 4). The lowest prevalence of MetS and that of most of the individual components of the MetS were observed in for the population living in the Amazon (26.6% for MetS) (Tables 2, 3 and 4). In contrast, the Amazon region had the highest prevalence of low HDL (66.6%) of all regions, mainly among women living in the Amazon (75.7%). Due to the unexpected high prevalence of low HDL-cholesterol, a correlation between cholesterol and HDL levels was performed in the amazon population. There was a positive correlation between total cholesterol and HDL-cholesterol only in women (Fig. 3).

### Overweight and MetS by economic status

**Fig. 4:**
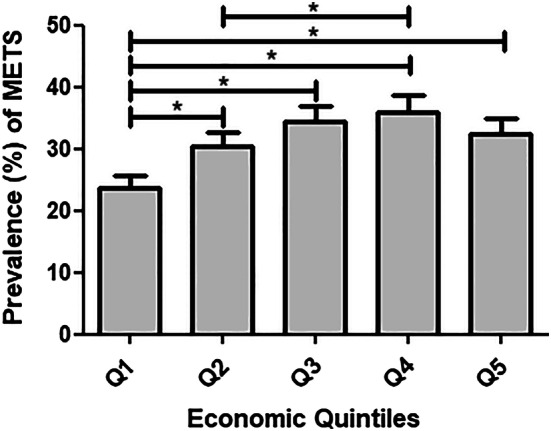
Mets by the socioeconomic status. *Significant differences between groups (*p* < 0.05)

The lowest quintile (Q1) of economic status had the lowest prevalence of overall MetS (Table 2), and of overweight or obesity and each of the individual MetS components (Table 3). As presented in Table 2 and Fig. 4, there is a gradient of MetS by economic status, where Q4 showed the highest prevalence. When comparing SES and MetS stratified by gender, we see that among men, Q1 showed the lowest prevalence but differences between the other quintiles (Q2–Q5) were generally small. In women, MetS showed a lower prevalence in Q1 but also in quintile Q5, compared to the other quintiles (Fig. 4 and Table, Online Resource 2, which describes the age-standardized prevalence of the individual components of the metabolic syndrome and overweight and obesity by area, altitude, region, and socioeconomic quintiles and by gender).

### The dangerous triads

The dangerous triads were more prevalent in men (10.5 vs 5.6), urban population (5.8 vs 2.9), and at low altitude (5.9 vs 2.2 and 3.2), whereas the lowest prevalence was in the amazon region (1.8 vs 3.4 Highlands, 6.8 Coast, and 7.1 Galapagos) and in the lower socioeconomic quintile Q1 (4.6 vs 8.2 Q4, or 8.1 Q5) (see Table, Online Resource 3, which shows the crude and age-standardized prevalence of the dangerous triads).

## Discussion

We showed that MetS is present overall in more than 30% of the adult population of Ecuador between 18 and 59 years old, and that around 85% of the population has at least one metabolic abnormality defined as either altered glucose metabolism, dyslipidemia, high blood pressure, or abdominal obesity. The prevalence of overweight, obesity, and MetS triple with age, where the prevalence from 18 to 29 is around 16% and increases to approximately 50% at the age group 50–59. Abdominal obesity and low HDL-cholesterol are the most common metabolic abnormalities in Ecuador, with approximately 65% and 60%, respectively. Besides highlighting the difficult public health problem that the overweight and MetS represent in Ecuador, in this study, we address in this study significant differences between populations that should be taken into consideration to establish population-based strategies to combat this serious health problem with strong economic implications.

The high prevalence of overweight and obesity in the general Ecuadorian adult population is particularly important in the Galapagos and the Coast regions. These regions also had around twice the prevalence of the dangerous triads. This finding in coastal areas is puzzling, since the diet is rich in sea food [20], which is considered to be a beneficial factor preventing the MetS [21]. Apparently other lifestyle factors (e.g., a high consumption of excess sugar and processed foods, a high intake of carbs and salt, and a sedentary and urbanized lifestyle in coastal cities) overrule the beneficial diet rich in sea food. Consequently, the coast and Galápagos regions have the highest risk of morbidity and mortality of the Ecuadorian population [6]. Preventive efforts should be mainly focused on reducing the blood pressure, highly prevalent in these regions, which is a key risk factor for a CVD event.

The population living in the Amazon region had the lowest prevalence of the individual metabolic syndrome MetS components, except for low HDL-cholesterol for which this region had the highest prevalence among all the altitudes. Since the Amazon region is less urbanized, we may speculate that a more rural lifestyle may have a beneficial effect to prevent the development of the MetS. Consequently, a more active lifestyle using a diet low in cholesterol and based on natural ingredients without usage of excess sugar and processed foods plays a role in MetS low prevalence in the Amazon area. The correlation of low total cholesterol with low HDL-cholesterol levels found in the Amazon population (be it only in women) could then also explain the high prevalence of low HDL-cholesterol, since cholesterol is necessary in the synthesis of HDL [22]. Another possible mechanism of this special lipidemic profile of the Amazon indigenous population could be a genetic variation causing lower HDL-cholesterol levels, as has been described previously in the literature [23].

Besides the differences in regions, this study showed a higher prevalence of MetS and overweight in urban compared to rural areas. This is in agreement with evidence from the other studies on a higher prevalence in urban areas, in both developed and developing countries [13, 24]. However, a recent publication on Iranian population described an increased prevalence of MetS and its components in rural compared to urban areas [25]. Inadequate physical activity, higher intake of sugar-enriched beverages and fast food, and low intake of fruit and vegetables are some factors that take part in urbanization lifestyle that account for the high prevalence of MetS and its components found in the urban population.

**Fig. 5:**
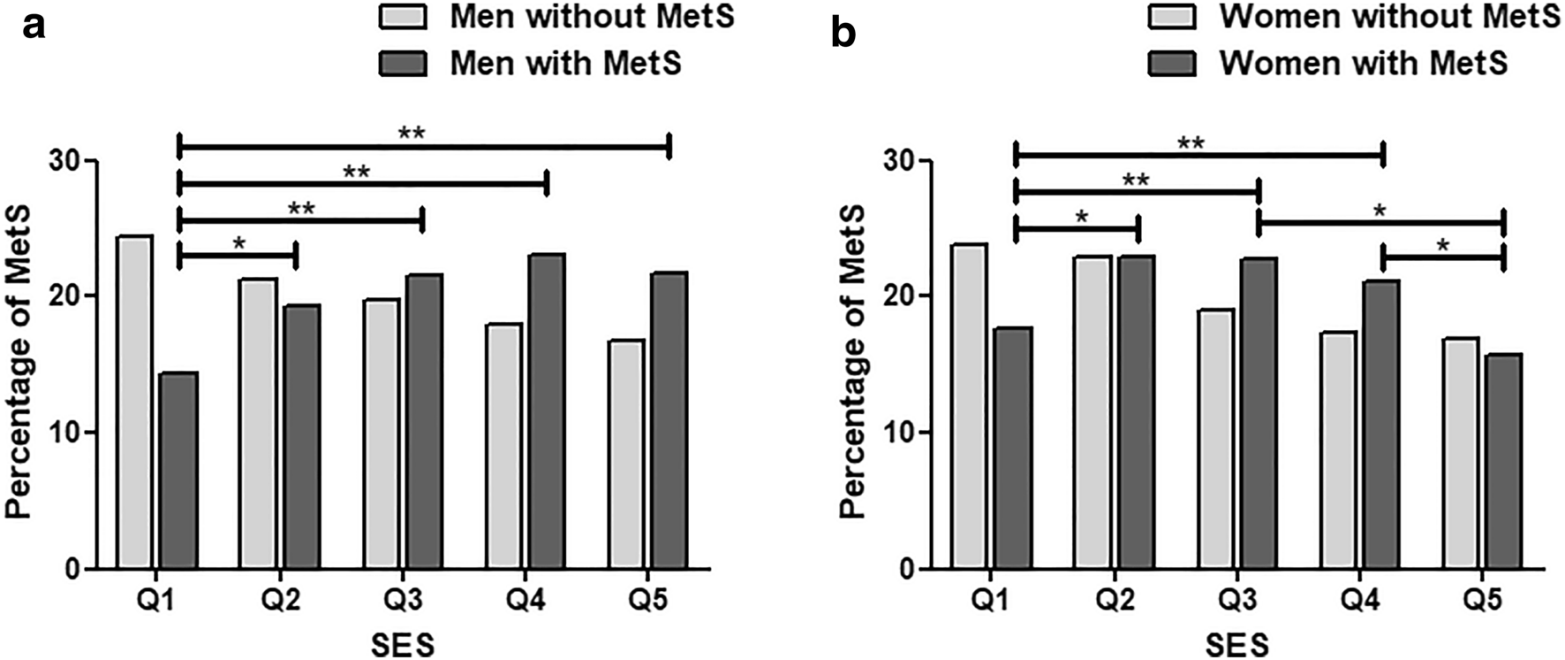
Mets in women and men by the socioeconomic status. **a** In men and **b** in women. *p < 0.05, **p < 0.001

With respect to socioeconomic status, the population with the lowest socioeconomic status had the lowest prevalence of MetS and almost all the individual components, while the high-income population had the worst metabolic profile. A similar SES pattern was found in a nation-wide study performed in Mexico [26]. In our study, however, women in the highest and lowest socioeconomic quintiles both had similar low metabolic risks, whereas for men, only the lowest socioeconomic status was associated with better metabolic health (Fig. 5). This crucial difference in SES between women and men was not reported in the Mexican population [26]. In this study, only men showed the characteristic pattern of undeveloped countries where belonging to a high-income class is correlated to the development of MetS [27, 28], while women agreed with a protective effect of high social class to MetS described elsewhere [29–32]. A difference between men and women in this respect has been reported previously [33, 34]. It is important to highlight that in developed countries, there is a transition where the higher socioeconomic class is reducing their metabolic risk, while it is increasing in lower social classes [32, 35], likely illustrating the adoption of a healthier lifestyle in the more effluent classes. We hypothesize that this change is already happening in Ecuador, since the women in the highest socioeconomic class showed a lower prevalence of MetS compared to the high–middle class which showed the highest prevalence. Unequal health access and social benefits will hamper the possible positive effects of preventive government measures to the population with the low income and middle income, resulting in a shift of better health in higher rather than lower socioeconomic groups.

In Latin America, there are a few nationally representative surveys addressing cardiometabolic risk factors [7, 8]. Obesity, high blood pressure, dyslipidemia, and diabetes are major public health problems globally. Despite this, there is still not enough information on the variation of the prevalence between and within countries and on the changes of these risk factors in time. To better understand this global pandemic, it will be advisable to know the differences between populations that will result in more focused research and directed policies to reduce the enormous problem that these non-communicable diseases represent. Additionally, the prevalence of risk factors is increasing in low- and middle-income countries, while in high-income countries, they have been suggested to have reached a plateau [36]. Consequently, as overweight and MetS increases, the risk of diabetes and cardiovascular disease morbidity and mortality will increase in parallel in these low- and middle-income countries [37–41]. Although, the lifestyle differences described above can explained partially the results, which is not clear how much these differences account for the demographic variations in the prevalence of the MetS, and overweight and obesity, and more importantly how they can be modified.

### Strengths and limitations

Strengths of our study include that the ENSANUT-ECU study was performed in a very large-scale nation-wide sample across the entire Ecuadorian country, using standardized protocols, instruments, and biomarkers, which enables the exploration of the heterogeneity across age, gender, and geographic and socioeconomic groups. Strict training and supervision were performed to ensure adequate data collection. The results thus offer exceptionally informative evidence on the prevalence of MetS and the sociodemographic determinants of the Ecuadorian society. An important limitation regarding the prevalence of metabolic syndrome is that the study did not include the population older than 59 years. This means that the found MetS prevalence is probably relatively low, since prevalence increases with aging. Another limitation is that we only measured all participants at one point in time and we cannot, therefore, provide evidence for changes in time within persons or generations.

## Conclusions

In conclusion, our results showed a high prevalence of overweight, obesity, and the MetS in Ecuador, and that there are crucial differences between region, area, socioeconomic status, and importantly by gender within the Ecuadorian population. Particularly, urban-dwelling men in the coastal regions of high socioeconomic state are at high risk to develop the MetS, while indigenous individuals living in the Amazon region are at the lowest risk. How this will impact further development of CVD in these separate populations need to be studied. Also, further studies evaluating the impact of diet, physical activity, socioeconomic status, and education could help to identify the factors responsible for the differences between gender, areas, and regions. Furthermore, the described prevalence of different metabolic risk factors for each region will allow to develop more targeted prevention strategies; for example, a diet strategy in Ecuador will be totally different between the Amazon region where it might be necessary to increase the HDL levels than for the coastal region where focus would be on reducing, in particular, the blood pressure.

## Electronic supplementary material

Below is the link to the electronic supplementary material.

Supplementary file1 (DOCX 21 kb)

Supplementary file2 (DOCX 22 kb)

Supplementary file3 (DOCX 18 kb)

## Data Availability

The data are available online at the web page of the Ecuadorian Health Ministry.
