# Supplementary material for: Prevalence of overweight and metabolic syndrome, and associated sociodemographic factors among adult Ecuadorian populations: the ENSANUT-ECU study
Source: J Endocrinol Invest. 2020 May 19;44(1):63–74. doi: 10.1007/s40618-020-01267-9 (PMC7796886; doi:10.1007/s40618-020-01267-9)
Supplement: Supplementary file 1 — Supplementary file1 (DOCX 21 kb) [file 40618_2020_1267_MOESM1_ESM.docx]

**Prevalence of overweight and metabolic syndrome, and associated sociodemographic factors among adult Ecuadorian populations: The ENSANUT-ECU study**

**Journal of Endocrinological Investigation**

Jorge Pérez-Galarza^1,2^, Lucy Baldeón^1^, Oscar H. Franco^2^, Taulant Muka^2^, Hemmo A. Drexhage^3^, Trudy Voortman^2^, Wilma B. Freire^4^.

^1^ Instituto de Investigación en Biomedicina, Universidad Central del Ecuador, Quito - Ecuador

^2^ Department of Epidemiology, Erasmus University Medical Center Rotterdam, the Netherlands.

^3^ Department of Immunology, Erasmus University Medical Center Rotterdam, the Netherlands.
^4^ Instituto de Investigación y Nutrición, Universidad San Francisco de Quito, Quito – Ecuador.

**Corresponding author**:

Jorge Pérez-Galarza, MD, MSc.

[jmperez@uce.edu.ec](mailto:jmperez@uce.edu.ec)

+593 992734290

Instituto de Investigación en Biomedicina (INBIOMED)

Universidad Central del Ecuador

Capitán Giovanni Calles. Hospital Docente de Caderón, Quito, Ecuador

ORCID ID: https://orcid.org/0000-0003-2742-3727

**Online Resource 1.** Table of age-standardized prevalence of the accumulative risk factors of the metabolic syndrome by subregion, area, altitude, region, and socioeconomic quintiles and by gender.

| **Accumulative risk factors of the metabolic syndrome** | | | | | | |
| --- | --- | --- | --- | --- | --- | --- |
|  | **0** | **1** | **2** | **3** | **4** | **5** |
| *Men* | | | | | | |
| *Urban* | 21.3 (19.5-23.1) | 21.5 (19.7-23.4) | 21.2 (19.3-23.1) | 20.3 (18.5-22.1)^‡^ | 12.7 (11.3-14.2)^‡^ | 3.0 (2.3-3.7) |
| *Rural* | 32.6 (29.4-35.7)^ỻ^ | 25.0 (22.3-27.7) | 17.3 (15.0-19.6) | 14.5 (12.4-16.5) | 8.6 (7.0-10.2) | 2.1 (1.3-2.9) |
| *0-500* | 23.1 (20.8-25.4) | 22.1 (19.8-24.4) | 19.0 (16.9-21.1) | 19.5 (17.4-21.7) | 12.9 (11.1-14.6)^±^ | 3.4 (2.5-4.3)^π^ |
| *501-1500* | 30.1 (25.7-34.5)^¶^ | 22.4 (18.6-26.2) | 18.9 (15.4-22.3) | 18.0 (14.6-21.4) | 9.7 (7.2-12.2) | 1.0 (0.2-1.8) |
| *>1500* | 27.0 (24.3-29.6) | 24.7 (22.2-27.3) | 20.4 (18.1-22.7) | 16.6 (14.5-18.6) | 9.2 (7.6-10.7) | 2.2 (1.4-2.9) |
| *Women* | | | | | | |
| *Urban* | 10.5 (9.5-11.5) | 24.1 (22.6-25.6) | 33.0 (31.3-34.7) | 20.2 (18.9-21.5)^‡^ | 9.3 (8.4-10.3) | 2.9 (2.3-3.4)^‡^ |
| *Rural* | 9.7 (8.5-10.9) | 27.7 (25.7-29.8)^ỻ^ | 34.6 (32.2-36.9) | 19.6 (17.9-21.3) | 7.3 (6.2-8.4) | 1.1 (0.7-1.5) |
| *0-500* | 13.6 (12.3-15.0) | 13.1 (11.7-14.4) | 11.3 (10.0-12.5) | 11.5 (10.3-12.8)^±^ | 7.6 (6.6-8.6)^κ^ | 2.0 (1.5-2.5)^π^ |
| *501-1500* | 14.7 (12.6-16.9) | 11.0 (9.1-12.8) | 9.3 (7.6-11.0) | 8.9 (7.2-10.5) | 4.7 (3.5-6.0) | 0.5 (0.1-0.9) |
| *>1500* | 14.8 (13.3-16.2) | 13.5 (12.1-14.9) | 11.2 (9.9-12.4) | 9.1 (7.9-10.2) | 5.0 (4.2-5.9) | 1.2 (0.8-1.6) |
| *Men* | | | | | | |
| Highland | 26.9 (24.5–29.4)^α^ | 23.6 (21.3–25.9) | 20.5 (18.4–22.7) | 16.6 (14.7–18.5) | 10.0 (8.5–11.5) | 2.3 (1.6–3.0) |
| Coast | 21.5 (18.8–24.2) | 22.5 (19.7–25.2) | 19.7 (17.1–22.2) | 20.0 (17.4–22.6) | 13.1 (10.9–15.2)^+^ | 3.3 (2.2–4.3)^+^ |
| Amazon | 31.1 (27.0–35.3)^α^ | 23.8 (20.2–27.4) | 17.1 (14.0–20.1) | 18.5 (15.3–21.7) | 8.2 (6.1–10.3) | 1.3 (0.4–2.1) |
| Galapagos | 16.5 (8.9–24.1) | 19.3 (11.0–27.5) | 18.3 (10.3–26.4) | 20.2 (11.8–28.6) | 19.3 (11.0–27.5)^+^ | 6.4 (1.7–11.2) |
| *Women* | | | | | | |
| Highland | 11.3 (10.1–12.4)^+^ | 25.8 (24.1–27.6)^β^ | 32.4 (30.4–34.4)^+^ | 21.3 (19.7–23.0) | 7.5 (6.5–8.4) | 1.7 (1.2–2.1) |
| Coast | 9.9 (8.5–11.4) | 24.3 (22.0–26.6) | 31.7 (29.1–34.2)^+^ | 20.4 (18.3–22.5) | 10.6 (9.1–12.1)* | 3.1 (2.3–3.9)* |
| Amazon | 8.3 (6.8–9.8) | 25.8 (23.1–28.4)^β^ | 39.9 (36.7–43.2) | 19.6 (17.3–21.9) | 5.4 (4.2–6.6) | 1.0 (0.5–1.5) |
| Galapagos | 8.0 (4.2–11.8) | 16.9 (11.4–22.4) | 35.2 (27.2–43.2) | 24.0 (17.4–30.5) | 15.0 (9.8–20.2)* | 0.9 (-0.4–2.2) |
| *Men* | | | | | | |
| Q1 | 36.0 (31.8-40.2)^ϕ^ | 27.5 (23.8-31.2)^¥^ | 15.1 (12.4-17.8) | 13.4 (10.9-16.0) | 6.8 (5.0-8.6) | 1.2 (0.4-1.9) |
| Q2 | 27.2 (23.5-30.9) | 23.1 (19.7-26.5) | 20.2 (17.0-23.4) | 16.6 (13.7-19.5) | 11.5 (9.1-13.9)^‼^ | 1.5 (0.6-2.3) |
| Q3 | 23.4 (19.9-26.9) | 23.8 (20.3-27.3) | 19.3 (16.1-22.4) | 20.1 (16.8-23.3)^‼^ | 11.4 (8.9-13.8)^‼^ | 2.1 (1.1-3.2) |
| Q4 | 21.6 (18.2-25.0) | 19.9 (16.7-23.2) | 21.3 (18.0-24.7)^‼^ | 19.7 (16.4-22.9)^‼^ | 13.3 (10.6-16.0)^‼^ | 4.2 (2.7-5.6)^Δ^ |
| Q5 | 19.5 (16.2-22.8) | 20.8 (17.4-24.3) | 22.3 (18.7-25.9)^‼^ | 21.4 (17.9-24.9)^‼^ | 11.9 (9.3-14.5)^‼^ | 4.0 (2.5-5.5)^Δ^ |
| *Women* | | | | | | |
| Q1 | 11.0 (9.3-12.7) | 28.8 (26.0-31.5)^з^ | 35.4 (32.4-38.5) | 17.5 (15.3-19.6) | 6.0 (4.7-7.2) | 1.3 (0.7-1.9) |
| Q2 | 9.2 (7.6-10.7) | 25.4 (22.9-27.9) | 34.6 (31.7-37.6) | 20.9 (18.6-23.2) | 8.0 (6.5-9.4) | 1.9 (1.2-2.6) |
| Q3 | 9.1 (7.5-10.8) | 22.0 (19.5-24.5) | 34.1 (31.0-37.3) | 23.6 (21.0-26.2)^‼^ | 9.1 (7.5-10.8)^‼^ | 2.0 (1.3-2.8) |
| Q4 | 9.8 (8.0-11.5) | 23.4 (20.7-26.1) | 31.6 (28.4-34.7) | 23.3 (20.6-26.1)^‼^ | 9.1 (7.4-10.7)^‼^ | 2.8 (1.8-3.7) |
| Q5 | 11.9 (9.9-14.0) | 25.6 (22.6-28.6) | 33.2 (29.7-36.6) | 18.9 (16.3-21.4) | 8.9 (7.2-10.7) | 1.5 (0.8-2.3) |

Results are-age standardized rate (95% CI). Significant differences (p value <0.05) compared to urban^ỻ^, rural^‡^, 0-500^¶^,501-1500^π^, >1500^±^, 501-1500 and >1500^κ^, Galapagos^β^, Coast and Galapagos^α^, Amazon^+^, Highland and Amazon*, Q2 to Q5^ϕ^, Q3^з^, Q4^¥^, Q1 and Q2^Δ^, and to Q1^‼^.
