# Supplementary material for: Prevalence of overweight and metabolic syndrome, and associated sociodemographic factors among adult Ecuadorian populations: the ENSANUT-ECU study
Source: J Endocrinol Invest. 2020 May 19;44(1):63–74. doi: 10.1007/s40618-020-01267-9 (PMC7796886; doi:10.1007/s40618-020-01267-9)
Supplement: Supplementary file 2 — Supplementary file2 (DOCX 22 kb) [file 40618_2020_1267_MOESM2_ESM.docx]

**Prevalence of overweight and metabolic syndrome, and associated sociodemographic factors among adult Ecuadorian populations: The ENSANUT-ECU study**

**Journal of Endocrinological Investigation**

Jorge Pérez-Galarza^1,2^, Lucy Baldeón^1^, Oscar H. Franco^2^, Taulant Muka^2^, Hemmo A. Drexhage^3^, Trudy Voortman^2^, Wilma B. Freire^4^.

^1^ Instituto de Investigación en Biomedicina, Universidad Central del Ecuador, Quito - Ecuador

^2^ Department of Epidemiology, Erasmus University Medical Center Rotterdam, the Netherlands.

^3^ Department of Immunology, Erasmus University Medical Center Rotterdam, the Netherlands.
^4^ Instituto de Investigación y Nutrición, Universidad San Francisco de Quito, Quito – Ecuador.

**Corresponding author**:

Jorge Pérez-Galarza, MD, MSc.

[jmperez@uce.edu.ec](mailto:jmperez@uce.edu.ec)

+593 992734290

Instituto de Investigación en Biomedicina (INBIOMED)

Universidad Central del Ecuador

Capitán Giovanni Calles. Hospital Docente de Caderón, Quito, Ecuador

ORCID ID: https://orcid.org/0000-0003-2742-3727

**Online Resource 2.** Table of age-standardized prevalence of the individual components of the metabolic syndrome and overweight and obesity by area, altitude, region, and socioeconomic quintiles and by gender.

| **Individual components of the metabolic syndrome** | | | | | | |
| --- | --- | --- | --- | --- | --- | --- |
|  | **Abdominal Obesity** | **Hyper-triglyceridemia** | **Low HDL cholesterol** | **High blood pressure** | **Hyperglycemia** | **BMI >25 kg/m^2^** |
| Men | | | | | | |
| Urban | 51.4 (48.5-54.3)^ỻ^ | 43.3 (40.6-45.9)^ỻ^ | 49.6 (46.8-52.5)^ỻ^ | 28.6 (26.4-30.8) | 17.8 (16.1-19.6)^ỻ^ | 62.2 (59.0-65.3)^ỻ^ |
| Rural | 37.9 (34.5-41.2) | 32.4 (29.4-35.5) | 39.0 (35.6-42.4) | 24.5 (21.8-27.2) | 13.9 (11.8-16.0) | 48.6 (44.8-52.4) |
| 0-500 | 49.2 (45.8-52.6) | 40.0 (36.9-43.1) | 46.0 (42.7-49.3) | 30.2 (27.5-32.8)^¶^ | 21.7 (19.5-24.0)^‡^ | 58.1 (54.4-61.8) |
| 501-1500 | 42.0 (36.8-47.2) | 38.3 (33.4-43.3) | 48.5 (43.0-54.1) | 17.5 (14.2-20.9) | 11.3 (8.7-14.0) | 56.5 (50.5-62.5) |
| >1500 | 43.4 (40.1-46.8) | 39.2 (36.0-42.4) | 44.9 (41.5-48.3) | 25.2 (22.6-27.8)^¶^ | 10.1 (8.4-11.7) | 56.1 (52.3-60.0) |
| Women | | | | | | |
| Urban | 75.0 (72.4-77.6) | 27.3 (25.7-28.9) | 67.6 (65.1-70.1) | 15.7 (14.4-17.0) ^ỻ^ | 16.9 (15.6-18.2) ^ỻ^ | 66.8 (64.3-69.3) ^ỻ^ |
| Rural | 72.2 (68.9-75.6) | 24.3 (22.4-26.3) | 68.5 (65.2-71.8) | 12.8 (11.4-14.3) | 12.4 (11.0-13.8) | 60.8 (57.8-63.9) |
| 0-500 | 75.3 (72.1-78.5) | 25.0 (23.1-26.9) | 68.6 (65.5-71.7) | 15.9 (14.4-17.4)^¶^ | 19.6 (17.9-21.2)^‡^ | 66.3 (63.3-69.4) |
| 501-1500 | 74.9 (70.1-79.8) | 25.3 (22.5-28.1) | 73.7 (68.9-78.5) | 8.3 (6.7-10.0) | 10.9 (9.0-12.8) | 65.7 (61.1-70.2) |
| >1500 | 74.1 (70.8-77.3) | 27.5 (25.5-29.5) | 65.9 (62.9-69.0) | 13.6 (12.2-15.0)^¶^ | 11.0 (9.7-12.2) | 65.1 (62.1-68.2) |
| Men | | | | | | |
| Highland | 44.7 (41.6-47.9) | 39.6 (36.7-42.6) | 45.2 (42.1-48.4) | 25.2 (22.9-27.6)^+^ | 11.2 (9.6-12.8) | 56.2 (52.7-59.7) |
| Coast | 49.7 (45.6-53.8) | 40.5 (36.8-44.2) | 44.6 (40.7-48.5) | 31.2 (28.0-34.5)^*^ | 24.3 (21.5-27.2)^*^ | 58.1 (53.7-62.6) |
| Amazon | 39.7 (35.1-44.4) | 37.2 (32.6-41.7) | 48.1 (42.9-53.2) | 18.7 (15.4-21.9) | 9.0 (6.8-11.3) | 55.5 (50.0-61.1) |
| Galapagos | 59.6 (45.1-74.1)^+^ | 40.4 (28.4-52.3) | 58.7 (44.3-73.1) | 35.8 (24.5-47.0)^+^ | 31.2 (20.7-41.7)^*^ | 69.7 (54.0-85.4) |
| Women | | | | | | |
| Highland | 74.2 (71.2-77.2) | 27.8 (25.9-29.6) | 66.2 (63.4-69.0) | 13.5 (12.3-14.8)^+^ | 11.3 (10.1-12.5) | 65.4 (62.6-68.3) |
| Coast | 74.8 (70.8-78.7) | 24.5 (22.2-26.7) | 66.8 (63.0-70.5) | 18.4 (16.4-20.3)^*^ | 22.4 (20.2-24.5)^*^ | 65.8 (62.0-69.5) |
| Amazon | 74.3 (69.8-78.8) | 24.5 (22.0-27.1) | 75.7 (71.2-80.3)^¥^ | 7.1 (5.7-8.5) | 9.2 (7.6-10.8) | 64.5 (60.3-68.7) |
| Galapagos | 85.0 (72.6-97.4) | 24.4 (17.8-31.1) | 68.5 (57.4-79.7) | 15.5 (10.2-20.8)^+^ | 30.5 (23.1-37.9)^*^ | 77.5 (65.6-89.3) |
| Men | | | | | | |
| Q1 | 29.2 (25.4-33.0) | 28.0 (24.3-31.8) | 36.1 (31.9-40.3) | 23.9 (20.5-27.4) | 13.7 (11.1-16.3) | 42.9 (38.3-47.5) |
| Q2 | 43.9 (39.2-48.6)^ϕ^ | 37.5 (33.1-41.8)^ϕ^ | 44.1 (39.3-48.8) | 25.9 (22.2-29.5) | 15.2 (12.4-17.9) | 55.9 (50.6-61.3)^ϕ^ |
| Q3 | 47.3 (42.4-52.3)^ϕ^ | 41.0 (36.5-45.6)^ϕ^ | 48.4 (43.4-53.4)^ϕ^ | 26.3 (22.7-30.0) | 15.5 (12.7-18.3) | 58.6 (53.1-64.0)^ϕ^ |
| Q4 | 53.9 (48.5-59.2)^ϕ^ | 44.7 (39.9-49.6)^ϕ^ | 51.0 (45.8-56.2)^ϕ^ | 27.8 (24.0-31.7) | 18.1 (15.0-21.3) | 64.7 (58.8-70.5)^ϕ^ |
| Q5 | 56.8 (51.1-62.5)^Δ^ | 47.5 (42.3-52.7)^Δ^ | 51.6 (46.2-57.1)^ϕ^ | 26.8 (22.9-30.7) | 14.7 (11.8-17.6) | 65.2 (59.1-71.3)^ϕ^ |
| Women | | | | | | |
| Q1 | 69.7 (65.4-74.0) | 21.6 (19.2-24.0) | 66.6 (62.4-70.7) | 12.0 (10.2-13.8) | 12.7 (10.9-14.6) | 58.8 (54.9-62.8) |
| Q2 | 76.6 (72.2-81.0) | 25.5 (23.0-28.1) | 69.6 (65.4-73.8) | 13.4 (11.5-15.2) | 13.8 (12.0-15.7) | 66.2 (62.1-70.3) |
| Q3 | 77.2 (72.5-82.0) | 28.5 (25.7-31.4)^ϕ^ | 71.6 (67.1-76.2) | 14.4 (12.4-16.5) | 15.8 (13.7-17.9) | 67.2 (62.8-71.6) |
| Q4 | 77.1 (72.1-82.0) | 29.5 (26.4-32.5)^ϕ^ | 69.6 (64.9-74.2) | 14.5 (12.4-16.7) | 16.2 (13.9-18.4) | 70.5 (65.8-75.2)^ϕ^ |
| Q5 | 73.3 (68.3-78.4) | 25.9 (22.9-28.9) | 64.5 (59.7-69.2) | 13.8 (11.6-16.0) | 14.4 (12.1-16.6) | 67.1 (62.3-72.0) |

Results are-age standardized rate (95% CI). Significant differences (p value <0.05) compared to rural ^ỻ^, 501-1500^¶^, 501-1500 and >1500^‡^, Amazon^+^, Highland and Amazon*, Highland and Coast^¥^, Q1 and Q2^Δ^, and to Q1^ϕ^.
