# Supplementary material for: Prevalence of overweight and metabolic syndrome, and associated sociodemographic factors among adult Ecuadorian populations: the ENSANUT-ECU study
Source: J Endocrinol Invest. 2020 May 19;44(1):63–74. doi: 10.1007/s40618-020-01267-9 (PMC7796886; doi:10.1007/s40618-020-01267-9)
Supplement: Supplementary file 3 — Supplementary file3 (DOCX 18 kb) [file 40618_2020_1267_MOESM3_ESM.docx]

**Prevalence of overweight and metabolic syndrome, and associated sociodemographic factors among adult Ecuadorian populations: The ENSANUT-ECU study**

**Journal of Endocrinological Investigation**

Jorge Pérez-Galarza^1,2^, Lucy Baldeón^1^, Oscar H. Franco^2^, Taulant Muka^2^, Hemmo A. Drexhage^3^, Trudy Voortman^2^, Wilma B. Freire^4^.

^1^ Instituto de Investigación en Biomedicina, Universidad Central del Ecuador, Quito - Ecuador

^2^ Department of Epidemiology, Erasmus University Medical Center Rotterdam, the Netherlands.

^3^ Department of Immunology, Erasmus University Medical Center Rotterdam, the Netherlands.
^4^ Instituto de Investigación y Nutrición, Universidad San Francisco de Quito, Quito – Ecuador.

**Corresponding author**:

Jorge Pérez-Galarza, MD, MSc.

[jmperez@uce.edu.ec](mailto:jmperez@uce.edu.ec)

+593 992734290

Instituto de Investigación en Biomedicina (INBIOMED)

Universidad Central del Ecuador

Capitán Giovanni Calles. Hospital Docente de Caderón, Quito, Ecuador

ORCID ID: https://orcid.org/0000-0003-2742-3727

**Online Resource 3.** Table of crude and age-standardized prevalence of the dangerous triads. AO: abdominal obesity. HBP: High blood pressure. HG: Hyperglycemia. LHDL: low HDL-cholesterol. HTGD: Hypertriglyceridemia.

| **Triads** | | |
| --- | --- | --- |
|  | **AO;HBP;HG** | **LHDL;HBP;HTGD** |
| Crude | 6.4 (5.8-7.0) | 9.1 (8.4-9.7) |
| Gender | | |
| Men | 5.3 (4.5-6.1) | 10.5 (9.5-11.6)^¶^ |
| Women | 4.4 (3.9-5.0) | 5.6 (5.0-6.2) |
| Area | | |
| Urban | 5.8 (5.2-6.4)^ỻ^ | 8.2 (7.5-8.9)^ỻ^ |
| Rural | 2.9 (2.4-3.5) | 5.8 (5.0-6.5) |
| Altitude | | |
| 0-500 | 5.9 (5.2-6.6)^β^ | 7.8 (7.0-8.6)^α^ |
| 501-1500 | 2.2 (1.6-2.9) | 4.9 (3.9-5.9) |
| >1500 | 3.2 (2.6-3.7) | 6.7 (5.9-7.5) |
| Subregion | | |
| Highland | 3.4 (2.9-3.9)^+^ | 7.0 (6.2-7.7)^+^ |
| Coast | 6.8 (5.9-7.7)* | 8.3 (7.3-9.4)^+^ |
| Amazon | 1.8 (1.2-2.4) | 4.3 (3.5-5.2) |
| Galapagos | 7.1 (4.2-10.1)* | 7.8 (4.7-10.8) |
| Economic quintile | | |
| Q1 | 3.0 (2.3-3.7) | 4.6 (3.7-5.5) |
| Q2 | 3.8 (3.0-4.6) | 6.3 (5.3-7.3) |
| Q3 | 4.1 (3.3-5.0) | 7.6 (6.4-8.8)^‡^ |
| Q4 | 5.5 (4.5-6.6)^‡^ | 8.2 (6.9-9.5)^‡^ |
| Q5 | 4.6 (3.6-5.6) | 8.1 (6.8-9.5)^‡^ |

Results are-age standardized rate (95% CI). Significant differences (p value <0.05) compared to women^¶^, rural ^ỻ^, 501-1500^α^, 501-1500 and ˃1500^β^, Amazon^+^ or Highland and Amazon*, and to Q1^‡^.
